# Does Epiphytic Lichen Translocation Work? Methods, Outcomes and Future Perspectives

**DOI:** 10.3390/plants15071042

**Published:** 2026-03-27

**Authors:** Sonia Ravera, Marta Agostini, Elisabetta Bianchi, Renato Benesperi, Erika Bellini, Patrizia Campisi, Luca Di Nuzzo, Juri Nascimbene, Luigi Sanità di Toppi, Monica Ruffini Castiglione, Luca Paoli

**Affiliations:** 1 Department of Biological, Chemical and Pharmaceutical Sciences and Technologies (STeBiCeF), University of Palermo, Via Archirafi 38, 90123 Palermo, Italy; sonia.ravera@unipa.it (S.R.); patrizia.campisi@unipa.it (P.C.); 2 Department of Biology, University of Pisa, Via L. Ghini 13, 56126 Pisa, Italy; marta.agostini@biologia.unipi.it (M.A.); erika.bellini@unipi.it (E.B.); luigi.sanita@unipi.it (L.S.d.T.); monica.ruffini.castiglione@unipi.it (M.R.C.); 3 Department of Biology, University of Florence, Via La Pira 4, 50121 Florence, Italy; e.bianchi@unifi.it (E.B.); renato.benesperi@unifi.it (R.B.); 4 Department of Biological, Geological and Environmental Science, University of Bologna, Via Irnerio 42, 40126 Bologna, Italy; luca.dinuzzo2@unibo.it (L.D.N.); juri.nascimbene@unibo.it (J.N.)

**Keywords:** assisted relocation, conservation translocation, endangered lichens, reintroduction, reinforcement

## Abstract

Epiphytic lichens are highly sensitive components of forest ecosystems, increasingly threatened by habitat disturbance and climate change. While habitat protection remains central to lichen conservation, translocation has emerged as a promising tool to address population decline, although its global effectiveness remains poorly evaluated. This scoping review, conducted under PRISMA-ScR guidelines, analyzes 30 taxa across 12 countries to evaluate current methodologies and outcomes. The reviewed literature is largely characterized by small-scale, method-oriented interventions, with a strong predominance of thallus fragment translocation over diaspore-based approaches. Success is most often evaluated through short-term survival and persistence of transplanted material, whereas indicators of long-term population self-maintenance and reproductive viability are rarely considered. Major limitations emerge from technical constraints, including early sample loss due to inadequate fixation, as well as from mismatches between donor requirements and recipient-site microhabitat conditions. Although high initial survival is frequently reported, evidence for long-term population stability, secondary colonization, and genetic resilience remains scarce. Overall, translocation may support short-term establishment under favorable environmental conditions, mainly at local scales, but its reliability as a long-term conservation strategy requires further validation. This review identifies a critical gap in long-term monitoring and highlights the need for research priorities that enhance the effectiveness, conceptual clarity, and technical precision of future translocation efforts to ensure the persistence of epiphytic lichen populations within changing forest landscapes.

## 1. Introduction

Lichens are complex multispecies symbioses, primarily involving a fungal partner (mycobiont) and one or more photosynthetic partners (photobionts), usually green algae or cyanobacteria [1]. Their biological traits underpin essential ecological functions in terrestrial ecosystems: they are pioneer colonizers of newly available substrates, contribute to soil stabilization and nutrient and carbon cycling, and provide microhabitats for diverse organisms [2,3]. Conserving the lichen biota is therefore essential not only for sustaining ecosystem processes but also for maintaining biodiversity at both local and regional scales. However, lichens are particularly sensitive to environmental change. Their vulnerability arises from the physiologically integrated yet structurally simple organization of the thallus, poikilohydric physiology, dependence on atmospheric sources for water and nutrients, slow growth rates, and generally limited dispersal and establishment capacity [4,5]. Atmospheric pollution, especially nitrogen deposition, and habitat disturbance are among the most pervasive threats, impairing photosynthetic performance, altering symbiotic balance, and reducing habitat suitability [6,7,8,9,10]. In parallel, climate change is modifying temperature and precipitation regimes, increasing the risk that stenotopic species (i.e., species with narrow ecological tolerance) will exceed their physiological tolerance limits [11,12,13]. These pressures often act synergistically, leading to population decline and local extinctions, particularly in epiphytic lichens dependent on long habitat continuity.

While in situ conservation through habitat protection remains a cornerstone of lichen conservation, it may be insufficient under habitat loss, land-use intensification, and rapid climate change [4,14]. Modeling approaches suggest that habitat restoration alone may not guarantee the recovery of epiphytic lichen populations following severe landscape fragmentation [15]. In such contexts, conservation translocations, i.e., the deliberate movement of organisms for conservation purposes, are increasingly considered as complementary tools [15,16,17].

Translocations encompass reintroductions within the historical range, reinforcements of declining populations, and managed relocation to areas predicted to remain suitable under future environmental conditions [18,19]. Their success critically depends on an understanding of species’ biology, ecological requirements, and clearly defined conservation objectives.

Evidence from other groups provides a useful, though cautionary, framework. Large-scale syntheses on vascular plant translocations in Europe indicate that, despite their increasing application, outcomes remain highly variable and often disappointing in the long term [20]. Short-term survival is frequently low, many populations fail within a few years, and only a small proportion becomes self-sustaining. Failures are commonly linked to inadequate site selection, poor knowledge of species ecology, insufficient propagule numbers, and stochastic events.

Comparable challenges emerge from a limited body of literature on bryophytes and fungi. In bryophytes, translocations have been relatively rare and often experimental, constrained by strong microhabitat specialization, poikilohydry, and slow growth. However, some targeted interventions and substrate-based approaches have yielded encouraging results when microclimatic conditions closely matched those of donor sites [21,22,23,24]. In fungi, direct translocation remains controversial due to incomplete knowledge of life cycles, cryptic population structure, host specificity, and complex biotic interactions. Consequently, most conservation efforts still focus on habitat-level protection rather than organismal relocation [25,26].

Lichens combine many of the constraints identified in these groups within a single biological system: their obligate symbiotic nature, slow growth, frequent substrate specificity, and high sensitivity to microclimatic humidity and atmospheric chemistry impose additional challenges for conservation interventions. Reproductive strategy is also a critical factor: sexual reproduction via fungal spores requires the presence of compatible photobionts at the recipient site, whereas asexual propagation through fragments, soredia, or isidia enables the dispersal of pre-assembled symbiotic units. While vegetative propagules may favor short-term establishment, sexual reproduction is essential for maintaining genetic diversity and long-term adaptive potential [4,27]. Consequently, reproductive mode must be carefully considered when designing translocation programs. Equally important is a precise characterization of the ecological niche of target species. Successful establishment depends on matching multiple factors at recipient sites, including substrate type and chemistry, bark texture, light availability, microclimatic conditions, air quality, and the presence of suitable photobionts. Without detailed autecological information, translocation efforts risk failure or unintended ecological consequences. In Italy, this growing interest has recently translated into coordinated national initiatives, such as the project BioCon*Lobaria* [28], specifically aimed at developing and testing conservation strategies using *Lobaria pulmonaria* as a model species, through population reinforcement, habitat assessment and long-term monitoring. This project represents one of the few structured attempts to integrate experimental translocation within a broader conservation framework for epiphytic lichens at the national scale.

Although previous narrative syntheses have reviewed the historical development and practical feasibility of lichen translocation in general terms [29,30,31,32], a systematic and conservation-focused evaluation specifically addressing epiphytic species, methodological heterogeneity, and long-term demographic outcomes remains lacking.

Against this background, this review aims to (i) compile and critically assess published case studies on the translocation of epiphytic lichens; (ii) evaluate methodologies, propagule types, and recipient-site characteristics associated with success or failure; (iii) identify key biological and ecological factors influencing establishment and persistence; (iv) highlight knowledge gaps and research priorities to guide future research and conservation practices; (v) critically evaluate the validity of translocation as a long-term conservation tool, explicitly distinguishing between individual persistence and population-level success; and (vi) identify conceptual limitations in the definitions and metrics of “success” adopted in existing published case studies.

## 2. Results

### 2.1. Basic Biological and Geographical Information

The assembled dataset, derived from 24 papers, comprised 30 translocated lichen species recorded across 12 countries. With the exception of two species, each species was addressed in a single published article; the exceptions were *Leptogium saturninum* (two articles) and *L. pulmonaria* (eleven articles). Moreover, with specific regard to *L. pulmonaria*, one translocation case was first described and later updated in subsequent work [33,34], whereas another transplantation study was later complemented by a long-term follow-up [35,36]. Less than half of the species (47%) were classified as nationally threatened and marked with an asterisk, as detailed in Table 1.

**Figure 1:**
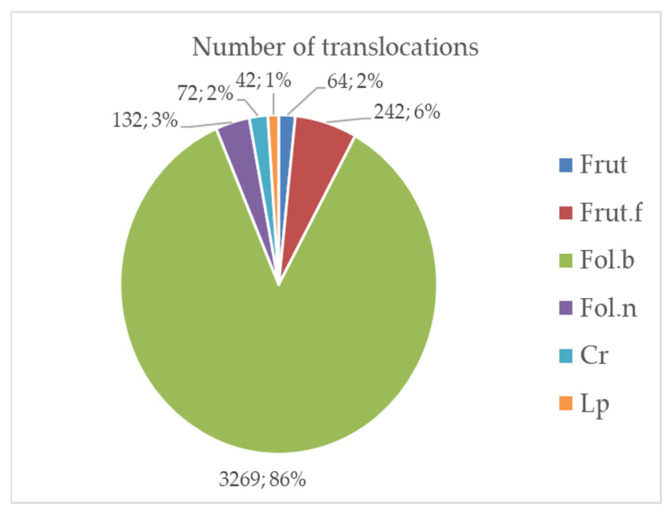
Number of epiphytic lichen translocations by growth form [53] (Cr: crustose, Lp: leprose, Fol.b: foliose broad-lobed, Fol.n: foliose narrow-lobed, Frut: fruticose, Frut.f: fruticose filamentous).

The overall dataset comprises around 5000 translocated samples (i.e., thalli, fragments, propagules or substrates), 4459 excluding records lacking explicit totals (e.g., ranges, “up to”, or qualitative estimates) [42,43,44,45,46]. Most of the translocated taxa were foliose lichens with broad lobes (86% of cases) (Figure 1).

**Figure 2:**
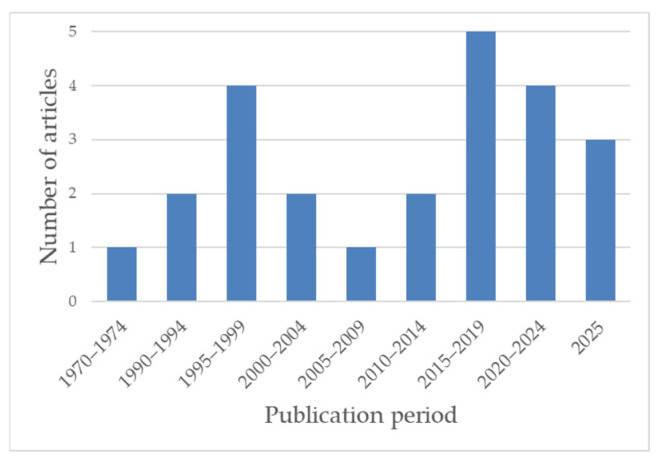
Temporal distribution of the selected papers, grouped into time intervals between 1970 and 2025.

Temporally, published translocation actions were unevenly distributed, with few early contributions and a marked increase in publication frequency from the mid-2010s onwards (Figure 2).

**Figure 3:**
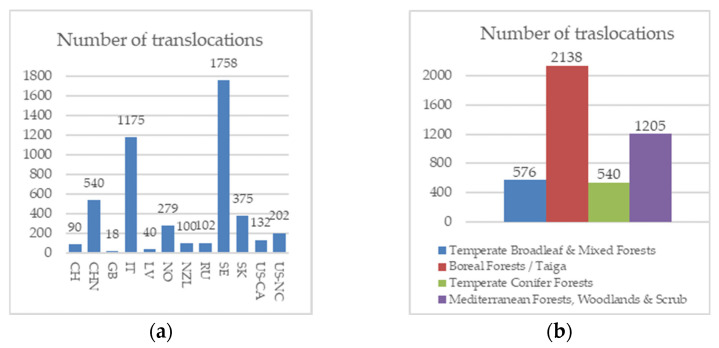
Distribution of epiphytic lichen translocation efforts across geographical, ecological and biological dimensions: (**a**) n. of translocations per country (codes according to ISO 3166 [57]); (**b**) n. of translocations across major biome types [58].

Among European countries, Sweden reported the highest number of translocations (1758, 37%), followed by Italy (1175, 24%) (Figure 3a). In Italy, several of these interventions were conducted at an experimental scale (e.g., [54,55,56]), addressing mainly technical and ecological aspects of translocation and contributing to the background for later conservation-oriented projects, such as BioCon*Lobaria* [28]. Most translocations were carried out in the Boreal Forests/Taiga Biome followed by Mediterranean Forests, Woodland and Scrub Biome, accounting for 47% and 27% of the cases, respectively (Figure 3b).

### 2.2. Experimental and Conservation Aims of Translocations

Most papers consist of conservation translocation experiments primarily aimed at population reinforcement [14,17,36,37,42,43,44,47,48,50]. A few describe conservation-driven reinforcement actions followed by long-term monitoring, but not based on a formal experimental design [34,52]. In a few cases [41,49], translocation was explicitly framed as a reintroduction, with lichens introduced into historically suitable but currently unoccupied sites to re-establish local populations. Another study tested two different practices simultaneously, combining population reinforcement at extant sites with attempted reintroduction into historically occupied areas [49].

A comprehensive PhD thesis further explored conservation translocation strategies across multiple epiphytic lichen species, explicitly combining population reinforcement and reintroduction approaches, with additional elements that can be interpreted as assisted colonization [42]. In another case, translocation was carried out into suitable but currently unoccupied restoration sites and can therefore be interpreted as assisted colonization [39]. Similarly, individuals were relocated from sites subject to imminent destruction into ecologically suitable but currently unoccupied restoration sites, an approach that can be interpreted as assisted colonization driven by impact mitigation rather than range expansion [38]. A further assisted colonization approach involved altitudinal translocation within the species’ range, adopting a space-for-time substitution to expose translocated thalli to climatic conditions representative of future scenarios [40].

Beyond population-focused translocations, some interventions have operated at the level of habitat or substrate rather than individual organisms. One large-scale example [59] involved the relocation of hundreds of deadwood substrates together with their associated multi-taxon communities within an ecological compensation framework, enabling the assessment of community-level responses. In another more targeted intervention [60], an entire living host tree bearing rare corticolous lichens was transplanted to prevent their loss due to infrastructure development. This mitigation-driven action focused on safeguarding a single substrate–community unit. Together, these cases highlight how conservation translocations may encompass entire habitat elements when species persistence is closely tied to their substrate.

Several papers also implemented experimental translocations to evaluate long-term survival under different deployment techniques and conservation-oriented forest management strategies [36,48].

Across all categories of intervention, translocations were overwhelmingly implemented at local or site-specific spatial scales. Most actions targeted individual populations, forest stands, or restricted sets of host trees, even when multiple recipient sites were involved within the same study. In these cases, sites were generally treated as independent experimental units rather than as components of a spatially connected population framework. Notably, no study explicitly adopted a landscape- or regional-scale translocation design aimed at enhancing population connectivity or long-term persistence across broader spatial extents.

### 2.3. Analysis of Translocation Practices

#### 2.3.1. Criteria for Selecting the Source Populations

When focusing exclusively on source population selection, five main criteria emerge across the papers: spatial proximity to the target site (same region/ecoregion, ecological continuity), minimization of donor impact (low-intensity harvesting, fragments, propagules), source material health, availability of suitable propagules or material (presence of thalli, diaspores, fragments usable for translocation), and conservation relevance of the donor population (belonging to rare/endemic/declining species). Most papers converge on a limited and recurrent set of criteria—namely spatial proximity, availability of suitable material, and conservation relevance of the species [14,34,39,42,43,44,47,48,49,59,60]. However, some contributions adopt partially divergent strategies [40,59]. In a few cases [34,41,49,50], source material was not collected from standing populations but recovered from thalli destined to be lost due to logging operations or from naturally fallen host branches, thereby avoiding additional pressure on donor populations. A comparable rationale underlies the translocation of entire host substrates and associated lichen communities from impact areas scheduled for destruction, where source selection is driven primarily by unavoidable habitat loss rather than by intrinsic donor population attributes [59,60]. A clear distinction emerges between the health of the donor population and the health of the source material. The condition of donor populations is rarely explicitly assessed or quantitatively characterized [36,45,59]. Nevertheless, several reinforcement actions—including highly localized interventions based on very small numbers of translocated units (e.g., [52])—implicitly assume donor population robustness, often inferred from long-term persistence and local abundance rather than from formal demographic or vitality assessments. By contrast, the physiological integrity and structural quality of the transplanted material are consistently emphasized across most actions and frequently represent the primary donor-related criterion in practical decision-making.

The recurrent selection of *L. pulmonaria* is consistently justified by its documented historical decline at the European scale (e.g., [36,47,49]) and/or its recognition as a threatened species at the national level (e.g., [14,36,38,44,45,46,47]), as well as by its established role as a characteristic old-growth forest indicator (e.g., [36,48]). In contrast, a few studies [37,40] adopted a markedly different experimental approach by selecting a pool of lichen taxa representing the principal growth forms (crustose, foliose, and fruticose) in order to test transplant techniques across a broader morphological and ecological spectrum.

#### 2.3.2. Criteria for Selecting the Recipient Site

Recipient site selection is generally informed by a recurring set of criteria: environmental suitability, referring to the matching of habitat and microhabitat conditions with species requirements, particularly in terms of humidity, light regime and forest structure; absence of major stressors, i.e., the exclusion of sites affected by strong anthropogenic disturbance; substrate availability and quality, including the presence, continuity and suitability of surfaces required for attachment and growth of the translocated material; spatial and ecological coherence, with recipient sites located within the same region or forest type as the source populations; and long-term persistence potential, reflecting the likelihood that a site can support viable populations over time under future environmental conditions.

While most studies [14,17,33,34,35,36,37,38,41,42,43,44,47,48,49,50,51,52,60] converge on subsets of these criteria, some adopt distinctive or partially divergent approaches. In certain cases, recipient sites are selected primarily on the basis of low anthropogenic impact, e.g., explicitly prioritizing the absence of atmospheric pollution over other habitat features [49]. Other approaches emphasize microclimatic contrasts linked to forest management (e.g., logged vs. unmanaged stands) [48,50], with limited consideration of long-term habitat stability. A further departure from species-centered site selection is represented by strategies focusing on the translocation of entire deadwood substrates and their associated communities [59], thereby prioritizing substrate diversity over fine-scale microhabitat matching for individual species. Several small-scale or mitigation-driven interventions, including early experimental studies and applied conservation actions (e.g., [43,44,52,60]), fall within this general framework; however, this reflects a primary emphasis on immediate establishment and loss prevention rather than on formalized site-selection planning. Finally, in one applied conservation-oriented framework, recipient sites were selected not only to ensure current establishment success but also to anticipate future climatic conditions, with the aim of enhancing the long-term persistence and resilience of translocated populations [40].

#### 2.3.3. Translocation Material and Propagation Units

Current literature indicates that epiphytic lichen translocation is mainly centered on the use of thallus fragments, which represent the most commonly used propagation unit across the reviewed literature [17,34,36,37,40,46,48,49,50]. By contrast, the application of asexual diaspores, i.e., soredia [39] and isidioid soredia [14,47], is restricted to a lower number of conservation efforts [47]. The use of both soredia and thallus fragments for conservation-oriented transplantation had already been discussed in early applied work on *L. pulmonaria* [46], anticipating later experimental refinements [42,43,44]. Similarly, the use of whole thalli appears to be a niche approach, employed mostly for fruticose lichens [37,40,41,52]. An alternative but still uncommon strategy involves the translocation of the entire thalli together with fragments of their original phorophyte, thereby preserving the attachment interface and reducing mechanical disturbance [38,52,59]. A significant methodological difference is observed in two conservation actions [59,60], where the focus shifts from species-specific propagation to community-level translocation through the relocation of entire host substrates.

#### 2.3.4. Fixation Techniques

Several approaches have been used to fix samples onto host trees in translocation studies. Most commonly, physical retention methods rely on textile supports—such as medical gauze, cheesecloth, or burlap—or on synthetic substrates attached to the bark [14,37,43,44,47,48]. Standardized artificial carriers have also been used to control propagule placement [47].

Suspension-based systems—traditionally and predominantly applied to fruticose species [37,42]—have been implemented by tying thalli directly to host trees using nylon cords or monofilaments, without intermediate supports, so that the thalli hang as pendants in direct contact with the bark [40].

Propagule-based techniques have included gel-filled or impregnated gauze packets designed to enhance soredia immobilization [39]. Adhesive-based techniques have been applied either alone [52], such as synthetic epoxy resin [34], carboxymethyl cellulose used as glue [17], or carboxymethyl cellulose-based hydrogels used to enhance propagule immobilization [42] or combined with mechanical fixation. Examples of combined approaches include the use of epoxy resin together with nylon monofilament to reattach whole thalli or host branch sections to new substrates [59], as well as vegetable glue reinforced with nylon nets [49], or nylon nets stabilized with metal staples [36]. A comparable mesh-based configuration, in which individual thalli are attached to a plastic net with thread and the net is secured to branches with cable ties, has also been used in recent experimental transplants [41].

A further textile-based device (consisting of a sterilized bandage supported by a plastic net, with thallus fragments tied using cotton threads) has been employed in micro-transplant experiments [50], following an approach previously developed and repeatedly applied by the same authors [56].

Direct fixation of adult thalli or thallus fragments to the bark using metal staples, without intermediate supports, has been reported only rarely and exclusively in reinforcement contexts, notably for naturally detached thalli of *L. pulmonaria* [44] and for thallus fragments of *Sticta fuliginosa*, *L. saturninum*, and *Menegazzia terebrata* fixed directly to the bark in experimental reinforcement studies [43]. In other studies, however, the fixation technique was not explicitly specified [38].

#### 2.3.5. Scale of Interventions and Number of Translocated Samples

The scale of interventions varied widely among actions, with the number of translocated units ranging from as few as four units in highly localized reinforcement actions [52], fourteen units in early pioneering actions [33,34], and even a single host tree in a mitigation-driven whole-substrate relocation [60], to several hundred units in large-scale conservation actions [35,36,37,40,49,50,59]. Intermediate cases involved the translocation of a few tens of units within small-scale conservation actions [14,33,38,41,42,43,44,47,48,51].

Large interventions often involved the translocation of several hundred of fragments or entire thalli [35,36,49,50], whereas community-level approaches relocated hundreds of whole substrates within ecological compensation frameworks [59]. In contrast, smaller-scale experiments generally involved one hundred or fewer units and were primarily designed to test fixation techniques and early establishment processes. In some experimental studies, however, the scale of intervention cannot be expressed as a single absolute number of translocated units, as multiple propagule types and experimental approaches were involved and sample size was reported in relative or functional terms (e.g., numbers of supports, sites, or survival proportions) rather than as cumulative totals [42,43,44]. Overall, large-scale interventions (≥500 samples or substrates) were generally associated with structured conservation frameworks and supported by long-term monitoring (e.g., [36,49,59]), whereas studies involving fewer units focused mainly on methodological aspects or early establishment dynamics (e.g., [37,39]).

#### 2.3.6. Monitoring Design and Success Indicators

Although all the studies examined incorporate post-translocation monitoring protocols, a marked heterogeneity is observed in terms of duration, frequency, and indicators used to assess intervention success, reflecting a prevailing focus on early establishment phases rather than on long-term population dynamics. Most investigations adopt short- to medium-term monitoring schemes, generally ranging from a few months (e.g., [14,37,38,39,41,44,47]) to a maximum of five years [17,35,40,43,48,49,50,51,59,60]. Only a limited fraction of studies extend observations over multi-year timescales [33,34,36,42], leaving processes of demographic persistence largely undocumented. This variability is also reflected in monitoring frequency, which ranges from closely spaced surveys in the months immediately following translocation [17,37,39,43,44,47,48] to seasonal or annual assessments [17,34,37,38,40,42,52,59]. In some cases, monitoring does not follow a clearly defined schedule [59,60], while in others it appears to have been conducted only once, presumably at the end of the experimental translocation project [45,49,59].

In terms of biological parameters, survival and persistence of the translocated material represent the most universally adopted success criteria. Survival is typically quantified as the proportion of remaining thalli, fragments, or diaspores over time, often after one or multiple growing seasons, and constitutes the primary and most consistent proxy for translocation success. By contrast, growth and structural development appear less systematically addressed. When included, growth is frequently documented through thallus expansion, lobe formation, or biomass increase. For instance, growth rates of *L. pulmonaria* have been explicitly quantified in long-term experimental studies [14,47], and one paper [49] reported the development of new lobules and rhizines following translocation. In several cases, however, growth monitoring is primarily focused on attachment to the substrate, considered a prerequisite for establishment rather than a demographic parameter per se [14,42,43,44,48]. Growth assessment is sometimes restricted to specific taxa within multitaxon translocations, reflecting species-specific objectives and detailed growth patterns that were not uniformly addressed across all taxa [59]. In one case [40], biomass and thallus growth were explicitly measured. Notable exceptions to the general scarcity of structural monitoring are represented by early multi-year experimental studies that explicitly documented juvenile thallus development from vegetative diaspores, including the formation of anchoring hyphae and progressive lobe differentiation [43,44]. These studies provide rare, detailed insight into early ontogenetic stages following translocation.

Indicators related to health and physiological functioning are included only in a subset of studies. Chlorophyll fluorescence, ultrastructural integrity, and trace element accumulation have been evaluated, thereby allowing post-transplant physiological performance to be directly assessed [49]. Physiological acclimation following transplantation has also been examined through chlorophyll fluorescence parameters and apparent electron transport rates in *Erioderma pedicellatum* transplants [41]. In other cases, photosynthetic rates, pigment content, and carbon exchange under varying climatic conditions have been quantified, providing a mechanistic understanding of acclimation capacity [40]. In addition, modeling of photosynthetic activation and microclimatic suitability has been integrated to link habitat quality with physiological performance [42]. Through these approaches, indirect inference of vitality based solely on growth has been surpassed, and a more robust framework for evaluating adaptive responses has been established.

Reproduction and self-maintenance remain rarely investigated parameters. Although several studies documented juvenile development from diaspores and their persistence over multiple years [43,44], explicit demographic or reproductive metrics are generally absent. A partial exception is represented by studies in which reproductive shifts (e.g., apothecia production) have been examined within a translocation framework, thereby addressing life-history strategy adjustments as a potential indicator of long-term viability and the capacity to establish self-sustaining populations [40,41]. Overall, the scarcity of studies assessing reproductive output or recruitment highlights a significant gap in the literature regarding the formation of self-sustaining populations.

In several cases, vitality is represented through subjective or semi-quantitative scales, particularly in field-based experiments where structural or physiological measurements are not feasible, e.g., assessing transplant performance in different forest contexts, combining survival and qualitative vitality evaluation [48].

In addition to biological indicators, several studies incorporate technical or methodological performance as part of the monitoring outcomes. In these cases, the effectiveness and feasibility of different translocation techniques or substrates are explicitly assessed [37,39,44,48,59], and success is defined not only in biological terms but also operationally, highlighting the applied dimension of translocation efforts.

Finally, some studies adopt species- or life-stage-specific monitoring strategies, reflecting differentiated objectives within the same work. For example, survival may be assessed across all transplanted taxa, while growth is quantified only for selected target species [37], or indicators are tailored to life-stage-specific responses [47,50].

### 2.4. Outcomes and Critical Issues

#### 2.4.1. Patterns of Translocation Success

Analysis of the reviewed papers (Table 2) indicates that translocation success in epiphytic lichens is strongly structured by the type of material transferred and by the success indicator adopted. Rather than representing a binary outcome, success spans a continuum from short-term retention to multi-year persistence and, more rarely, documented physiological performance or stability. Here, “survival” and “retention” are treated as distinct indicators, reflecting physiological persistence and mechanical persistence, respectively.

While most studies declare success, only a subset provides explicit percentages relative to the translocated material, and fewer evaluate success beyond short-term survival. Short-term survival represents the most frequently declared outcome. Reported survival percentages vary widely depending on species, propagule type, fixation technique, and monitoring duration.

When whole adult thalli are translocated, short- to medium-term survival is generally high. Across studies, survival after 1–4 years typically ranges between approximately 71% and 100% [33,38,40,41,49,59]. Large-scale transplantation experiments involving more than 1000 thalli reported 89% survival after 20–25 months, whereas extended monitoring revealed a decline to approximately 23% after 14 years [35,36], demonstrating progressive attrition over time. Similarly, a 20-year reinforcement effort resulted in persistence of 43% of the original transplants [33,34]. These cases illustrate that high short-term survival does not necessarily translate into long-term demographic stability.

Translocation of thallus fragments shows greater heterogeneity. Fragment retention or establishment ranges from approximately 0% to 98%, depending on species, fixation method and environmental context [17,37,43,50]. In some papers, success was measured as simple fragment persistence [37,43], whereas in others, establishment required anchorage and renewed growth [42,47]. Growth-based metrics, including thallus area increase, were used in certain cases when survival percentages were not explicitly reported [44,50].

Propagule-based approaches frequently reported lower retention rates and more unstable outcomes. Reported retention or establishment values typically ranged between 10% and 60% [14,39,42,43,44]. In several experiments, initial retention declined markedly over time, highlighting the critical transition from mechanical attachment to biological establishment [14,43]. Successful differentiation into juvenile thalli was documented only in a limited number of multi-year studies [42,44], representing rare evidence of establishment beyond initial persistence.

Unsuccessful or partially successful outcomes were associated with inadequate fixation methods or high initial detachment rates [48,60]. Early loss phases were commonly followed by stabilization once anchoring hyphae developed, marking a transition from mechanical retention to establishment [14,44].

A more restrictive level of success involves documented thallus growth or establishment *sensu stricto*. Growth rates of *L. pulmonaria* were explicitly quantified in multi-year experiments [44,47], and growth probability linked to microclimatic alterations following forest management has been reported [50]. New lobule and rhizine formation following transplantation were also reported for *L. pulmonaria* [49], indicating active structural development.

Quantitative biomass and growth measurements under simulated climate change conditions revealed marked interspecific variability [40], with some species exhibiting enhanced growth and others declining. Suspension-based translocations also demonstrated measurable thallus expansion in *Usnea* species [37].

Beyond structural growth, a further and more stringent level of success assessment involves direct evaluation of physiological performance. While such approaches can provide useful information on post-transplant responses, their relevance depends on the specific objectives of the translocation, which may range from understanding the causes of failure to ensuring long-term demographic persistence. Physiological validation of translocation success remains relatively uncommon but can contribute to a more comprehensive interpretation of outcomes. In the few cases where it has been assessed, maintenance of chlorophyll fluorescence efficiency, preservation of ultrastructural integrity, and stability of trace element balance have been demonstrated following translocation [49]. Experimental transplants of *E. pedicellatum* further quantified post-transplant acclimation through chlorophyll fluorescence parameters and apparent electron transport rates, revealing heterogeneous physiological responses among transplanted thalli [41]. In addition, photosynthetic rates, pigment content, and carbon exchange responses have been quantified under varying or simulated climatic conditions, allowing evaluation of acclimation capacity [40]. Modeling approaches have also been applied to link photosynthetic activation and habitat suitability to microclimatic conditions, thereby connecting environmental context with functional performance outcomes [42].

These studies indicate that high survival percentages may coincide with sustained physiological functionality; however, physiological stability does not automatically translate into demographic persistence.

Explicit demographic validation is largely absent. Only one experimental framework explicitly incorporated reproductive output (e.g., apothecia production) as a success criterion [40]. Multi-year juvenile persistence [43,44,47] suggests potential for establishment, but without confirmation of self-sustaining population formation.

Community-scale translocations [59,60] assessed success in terms of assemblage stability and colonization dynamics rather than individual survival.

In several experimental contexts, technical or methodological performance constitutes an additional dimension of success. This includes large-scale substrate or habitat translocations aimed at conserving entire epiphytic communities, where success is evaluated in terms of species richness, assemblage stability and colonization dynamics rather than individual survival or growth [59]. Studies aimed at developing or comparing fixation techniques, artificial substrates or transplant devices often evaluated outcomes in operational terms, such as retention efficiency, durability of materials and feasibility under field conditions [17,37,39,42,44]. Although these outcomes do not directly reflect biological success, they provide essential methodological foundations for subsequent conservation-oriented applications.

#### 2.4.2. Critical Issues and Causes of Failure

Across the reviewed literature, failures in epiphytic lichen translocation were associated with a limited number of recurrent factors, although their relative importance varied among experimental contexts. One of the earliest documented translocation attempts, involving *L. pulmonaria*, resulted in complete mortality of the transplanted material [45], illustrating the vulnerability of early interventions conducted without formalized site-selection or fixation protocols.

Sample loss emerges as one of the most pervasive issues. Poor retention of propagules or fragments during the early post-translocation phase emerged as a major constraint, particularly when immobilization is insufficient. Inadequate fixation markedly reduces establishment success (e.g., [48]), whereas the use of adhesive-based immobilization systems substantially improves propagule retention [39]. Survival further depends on fragment size and attachment method, with smaller fragments generally showing higher persistence than larger ones, which in some cases exhibited very limited or no survival [17]. This problem was already highlighted in early experimental work [14,33,44], where a substantial proportion of transplants failed to remain attached to the substrate during the initial months, even under suitable conditions, indicating that fixation efficiency is a critical bottleneck during early establishment [42,43].

Closely linked to this, mechanical disturbance and fixation failure further contributed to losses, ranging from branch breakage due to heavy winter snowfall [40] and wind-related loss [38] to additional external factors, even exacerbated by the activity of large animals (e.g., bears [37]) or by fragmentation of transplanted thalli during experimental manipulation [41].

Biotic interactions were only rarely identified as direct causes of failure. In a limited number of cases, loss of thallus portions was attributed to invertebrate grazing, e.g., [17,34,39,47], less frequently including competitive overgrowth by epiphytic bryophytes, which in some cases reduced propagule persistence or delayed establishment [39,43,44]. A distinct type of biotic constraint was represented by the local decline of translocated thalli associated with the development of a lignicolous fungal infection on the host tree [52]. However, community-level translocation studies indicate more subtle constraints. In a large-scale deadwood translocation experiment [59], a stable overall lichen richness is reported over four years, but simultaneous colonization of generalist species and disappearance of specialized lignicolous taxa are also documented, suggesting that apparent stability may mask underlying compositional turnover and substrate-specific vulnerability.

Microclimate-related conditions consistently influenced translocation outcomes. Even when initial attachment was successful, subsequent development and survival were strongly affected by local microenvironmental conditions at the bark surface [14,41,42,43,44]. Reduced humidity, increased exposure, and changes in stand structure were associated with decreased growth and survival [35,50].

Marked differences in performance were also observed among host tree species, indicating strong interactions between substrate properties and microclimatic conditions [17]. Successful translocations were largely restricted to sites with high environmental quality, whereas transplants placed in marginal or disturbed environments frequently exhibited bleaching, necrosis, or detachment [42,49]. A comparable pattern of delayed decline was observed during long-term monitoring [52], which revealed site-dependent reductions in thallus vitality associated with biotic interactions and progressive environmental deterioration, despite initially successful establishment. Similarly, long-term monitoring of *L. pulmonaria* transplants over 14 years demonstrated a substantial cumulative decline in survival, with only 23% of transplants remaining despite high short-term survival [36]. These findings indicate that early post-translocation success may obscure progressive mortality driven by prolonged exposure to altered microclimatic conditions. Long-term observations extending over twenty years further showed that although most transplants of *Ricasolia amplissima* remained viable, some colonies experienced gradual late-stage reductions in thallus area due to bryophyte overgrowth and slow deterioration, and no spontaneous colonization of adjacent trees was recorded [34], pointing to limited secondary dispersal following transplantation. Consistent with these patterns, high survival (92% after 3.5 years) was reported for translocated *Sulcaria isidiifera*, together with marked variation in physiological performance depending on host species, aspect, and height above ground [51]. Comparable patterns of heterogeneous physiological performance despite overall survival have also been reported in experimental transplants of *E. pedicellatum* [41]. Together, these studies underscore that long-term translocation outcomes are strongly shaped by fine-scale microclimatic conditions and that initial establishment does not necessarily guarantee persistence.

Extreme weather events were not explicitly identified as direct drivers of failure in the reviewed studies. Although developmental dynamics were sometimes associated with favorable weather periods [39], failures were not directly linked to storms, droughts, or frost episodes.

Finally, unsuitable recipient site conditions clearly limited success. Translocation attempts consistently failed in sites affected by air pollution or heavy metal accumulation, despite evidence of initial physiological activity of the transplanted material [49]. Similarly, early multi-site translocation experiments showed that apparent habitat suitability based on the presence of adult thalli did not necessarily translate into successful establishment of transplanted propagules, underscoring the importance of fine-scale recipient site selection [44].

## 3. Discussion

### 3.1. Interpreting Translocation Success Across Studies

The reported success rates for epiphytic lichen translocation vary drastically, ranging from complete failure in early attempts [45] to survival percentages exceeding 90% in short-term assessments [33,38,41,59]. This variability does not necessarily reflect intrinsic differences in the biological feasibility of translocation. Rather, it primarily emerges from methodological heterogeneity and from divergent definitions of “success”.

Across the reviewed literature, success is most commonly defined as short-term survival or mechanical retention of the transplanted material [34,37,38,49]. In other cases, more restrictive criteria are adopted, including thallus growth, structural development, physiological performance, or early juvenile formation [17,39,40,41,43,44,47,49,59]. Because these indicators reflect distinct biological processes, similar percentages may represent fundamentally different conservation outcomes. In this context, it is important to distinguish between “survival” and “retention”, which, although sometimes used interchangeably, refer to different processes. Survival generally implies the persistence of physiologically functional thalli, whereas retention indicates the mechanical persistence of fragments or propagules on the substrate, without necessarily reflecting biological viability. Survival without growth, growth without reproduction, and reproduction without dispersal capacity correspond to different levels of ecological integration.

This definitional ambiguity represents not merely a methodological issue, but a conceptual limitation affecting the interpretation of conservation effectiveness. A similar pattern has been documented in vascular plant translocations, where short-term establishment often overestimates long-term viability [20]. Within lichens, the distinction between individual persistence and population-level success is rarely made explicit, despite being central to conservation translocation frameworks [18,19].

The contrast between diaspore-based and fragment-based approaches further illustrates this problem. Vegetative diaspores frequently show low establishment rates when success is defined as stable anchorage and early development [39,43,44]. In contrast, thallus fragments or entire thalli often exhibit high short-term survival, especially within limited monitoring times [35,40,41,49]. However, whole-thallus survival may partly reflect physiological inertia, as intact individuals bypass early establishment bottlenecks without necessarily achieving demographic integration. Without explicit differentiation between biological units and success criteria, cross-study comparisons remain intrinsically problematic.

A further source of variability originates from the diverse goals driving translocation interventions, as recognized in conservation translocation frameworks [18,19]. In reinforcement actions, the main purpose is usually to increase the size or improve the viability of existing populations; in such cases, success can often be reflected in measures of survival and short-term persistence. In contrast, reintroductions and assisted colonization aim to establish populations in areas where the species is currently absent, which requires clear evidence of reproduction, recruitment, and long-term persistence. Mitigation-driven translocations, on the other hand, tend to emphasize the immediate survival of individuals or communities displaced from sites affected by human activities, sometimes without ensuring their long-term demographic stability. These differences highlight the importance of defining success criteria a priori, since similar outcomes can reflect different levels of conservation effectiveness depending on the underlying objectives [18,19].

### 3.2. Biological Units, Species Sensitivity and Ecological Context

The type of biological unit translocated emerges as a primary driver of outcome. Vegetative diaspores represent the most vulnerable life stage, being highly sensitive to microclimatic instability and surface conditions during early establishment [43,44]. Substantial initial losses are common before anchoring structures develop and stabilization occurs. Even when early retention is low, such approaches may retain long-term conservation potential, as they minimize donor impact and preserve natural colonization dynamics [39]. However, an additional but rarely addressed dimension concerns the genetic and evolutionary implications of propagule choice. Translocation strategies based predominantly on vegetative fragments or isidioid diaspores may facilitate short-term establishment but potentially limit genetic recombination and adaptive potential, particularly if source material derives from a restricted number of donor individuals. Despite the recognized importance of sexual reproduction for maintaining genetic diversity and long-term resilience [4,27], almost no study explicitly evaluates post-translocation sexual regeneration or recruitment from ascospores. At the same time, many lichen species reproduce predominantly through asexual propagules and are able to persist in large and stable populations without clear evidence of ongoing sexual reproduction. However, even in these cases, some form of reproduction—sexual or vegetative—remains necessary to ensure long-term population maintenance and turnover.

Translocation of thallus fragments represents an intermediate strategy and the most frequently adopted approach in the literature [17,34,36,37,40,43,44,47,49,50]. Success varies markedly among taxa. Macrolichens transplanted as adult thalli or large fragments, such as *L. pulmonaria*, often show comparatively higher survival and measurable growth under favorable conditions [36,49], whereas more sensitive taxa, including cyanolichens, exhibit lower success even when similar techniques are applied [17,41,59]. These contrasts indicate that methodological refinement, while important, is insufficient when recipient site conditions do not adequately match species ecological requirements.

The highest apparent success rates are most often reported for the translocation of the entire thalli (Table 2), likely because intact individuals retain greater physiological integrity and bypass some of the critical bottlenecks associated with early establishment [34,37,44,59]. Nevertheless, this strategy entails higher ecological and ethical costs, as it requires the removal of substantial biomass from donor populations. Consequently, it is rarely appropriate for small, fragmented, or declining populations, which substantially limits its practical use in conservation.

At the community level, substrate-based approaches, such as deadwood translocation [59], highlight a shift in the definition of success: rather than focusing on the survival of individual thalli, these methods aim to promote the persistence and reassembly of epiphytic lichen assemblages as a whole.

Across all biological units, recipient-site quality remains decisive. Successful outcomes are largely confined to sites characterized by high humidity, low pollution levels, and structural continuity. Microclimatic alterations following logging have been shown to affect growth and vitality even in initially successful transplants [35,50]. Likewise, air pollution and trace element accumulation may impair physiological stability despite apparent survival [49]. These findings confirm that translocation success depends on fine-scale ecological matching rather than on broad habitat classification alone.

### 3.3. Technical and Temporal Bottlenecks in Assessing Success

Technical constraints and monitoring design strongly shape reported translocation outcomes. Early sample loss due to insufficient fixation is a pervasive issue, particularly for diaspores and small fragments, and has been repeatedly identified as a major bottleneck [37,39,43,44]. Improvements in fixation techniques enhance early retention, but do not guarantee long-term establishment if environmental conditions are suboptimal [17,49]. The contrast with early unsuccessful attempts [45] underscores the methodological progress, yet also highlights persistent structural limitations.

A further limitation concerns the temporal scale of monitoring. Most studies adopt short- to medium-term monitoring schemes, often limited to one or two years [39,40,41,44,46,47,50]. While early survival and physiological activity are necessary prerequisites for establishment, they do not provide evidence of population self-maintenance. Long-term observations illustrate this distinction clearly. For example, the 20-year monitoring of *R. amplissima* documented continued survival of several translocated thalli but provided no evidence of secondary colonization or population expansion [34]. Similarly, survival of *L. pulmonaria* declined substantially over 14 years despite high initial success [36]. These cases demonstrate progressive attrition and highlight the difference between individual persistence and demographic viability.

Indicators related to reproduction, secondary dispersal and recruitment remain rarely incorporated into monitoring protocols [39,43,44], and only exceptionally addressed explicitly [59]. Even when reproductive structures are documented [40], demographic consequences are seldom evaluated. Given the longevity and slow growth of many macrolichens, monitoring frameworks shorter than one decade may be insufficient to assess stability meaningfully.

This temporal bias creates what may be termed an “illusion of success”: short-term positive outcomes may mask long-term limitations driven by microclimatic stress, competition, or gradual physiological decline. Given the heterogeneity of study designs, these patterns should be interpreted as structural tendencies rather than statistically comparable outcomes, consistent with the exploratory scope of this review.

From a conservation perspective, these points highlight the importance of explicitly weighing the costs and benefits of translocation efforts. Such interventions demand considerable resources and can significantly affect donor populations, particularly when entire thalli or large fragments are removed. In this light, short-term indicators of success may give a misleading impression if they are not evaluated alongside ecological costs and long-term outcomes. The “illusion of success” highlighted in this review therefore reflects a possible mismatch between encouraging initial results and the true conservation value of the action. Incorporating cost–benefit assessments from the outset would enhance both the transparency and overall effectiveness of conservation translocations. In particular, the following aspects should be carefully considered:

- Impact on donor populations: removal of biomass (especially whole thalli) may compromise already vulnerable populations.
- Type of propagule used: balance between higher short-term success (whole thalli) and lower ecological impact (diaspores/fragments).
- Potential for long-term establishment: including evidence of reproduction and population persistence.
- Monitoring feasibility and duration: availability of long-term (>10 years) monitoring;
- Risk of “illusion of success”: use of short-term indicators only.
- Cost vs conservation value: comparison with alternative actions (e.g., habitat protection).
- Integration with broader conservation strategies: whether translocation complements or substitutes habitat conservation.

Overall, the drivers of translocation failure identified in this review can be grouped into three interacting domains: technical bottlenecks affecting early retention, microclimatic and substrate-related mismatches influencing performance across short- to medium-term timescales, and broader landscape-level constraints—such as habitat fragmentation, limited connectivity, and environmental degradation—affecting long-term demographic persistence (see Section 3.4). Importantly, these domains should not be interpreted as strictly separable, as mismatches operating at both local and landscape scales may jointly influence medium- and long-term outcomes. While the first two domains can be at least partially optimized through improved transplanting protocols and the careful selection of appropriate translocation units and microsites, landscape-level constraints are inherently more challenging to address. These depend on wider spatial processes and long-term habitat dynamics that extend beyond the scale of individual interventions and therefore require dedicated, large-scale and long-term investigations. Recognizing these hierarchical levels helps reconcile heterogeneous outcomes and clarifies that technical success does not necessarily imply ecological or demographic effectiveness.

### 3.4. Spatial Scale and Conservation Effectiveness

A recurring but rarely explicit issue emerging from the reviewed literature is the mismatch between the spatial scale of translocation interventions and the scale at which major threats to epiphytic lichens operate. Translocation studies are overwhelmingly conducted at local scales, often focusing on individual trees, forest patches, or single populations. While such designs allow experimental control, they inherently limit the capacity of translocation to address landscape-level drivers such as habitat fragmentation, declining air quality, or climate change. At the same time, translocations may, in some cases, contribute to maintaining demographic connectivity by establishing populations in previously unoccupied sites, although such objectives are rarely explicitly incorporated into study design or evaluated at broader spatial scales.

From a metapopulation perspective [15], local reinforcement may increase short-term occupancy without effectively supporting regional population dynamics or colonization processes. In fragmented landscapes, translocated populations risk functioning as demographic sinks if habitat continuity and environmental quality are not maintained at broader scales. Similar limitations have been identified in plant conservation translocations, where local successes do not necessarily translate into regional persistence [20].

In the absence of integrated landscape-level conservation, translocation may therefore produce spatially confined and potentially transient effects. Explicit incorporation of spatial scale into planning and evaluation—through connectivity analysis, identification of climate refugia, and long-term habitat protection—appears essential for assessing realistic conservation relevance [18,19]. In this context, translocations could potentially contribute to enhancing connectivity by acting as “stepping stones” between existing populations in fragmented landscapes, although this aspect remains largely unexplored in epiphytic lichens. Predictive modeling approaches may also help identify current and future climate refugia, allowing efforts to be directed toward areas with higher long-term habitat suitability and to experimentally assess species responses under changing climatic conditions. More broadly, translocations could be conceived not as isolated interventions but as part of coordinated strategies aimed at improving population persistence across landscapes, with monitoring schemes designed to capture spatial dynamics such as colonization and local extinction processes. Such approaches remain largely conceptual but may represent an important direction for future research.

## 4. Materials and Methods

**Figure 4:**
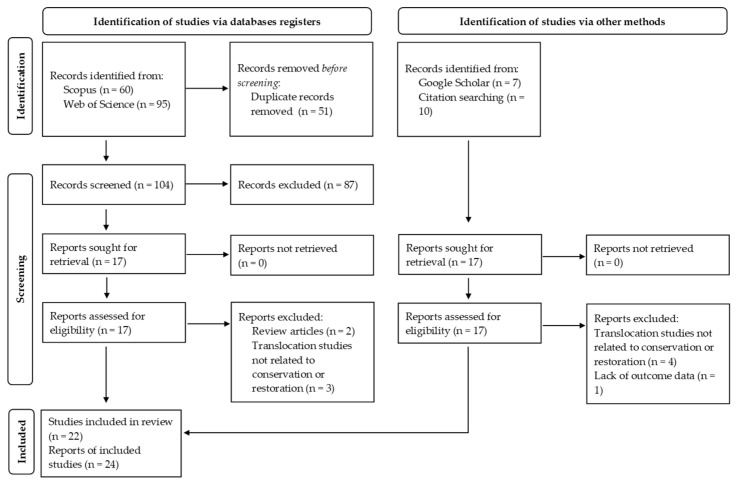
PRISMA 2020 flow diagram [61] for systematic reviews which included searches of databases and other sources.

We conducted a scoping review aimed at mapping the existing scientific literature on the conservation-oriented translocation of epiphytic lichens, with the objective of identifying temporal and geographical patterns, methodological approaches, and recurrent factors influencing translocation outcomes. The review was conducted following the PRISMA-ScR guidelines where applicable (Figure 4).

A systematic search of the scientific literature published in English was performed using Web of Science https://www.webofscience.com/wos/woscc/basic-search, (accessed on 2 January 2026), the databases Scopus (Elsevier) https://www.scopus.com/search/form.uri (accessed on 2 January 2026), and Google Scholar (https://scholar.google.com). Searches were conducted in January 2026, without initial restrictions on publication year.

The search strategy was intentionally focused on terminology commonly used in lichenological literature addressing conservation-related translocation practices. It combined terms referring to lichens (“lichen”, “lichens”, “lichenized fungi”), translocation or reintroduction approaches (“translocation”, “relocation”, “reintroduction”, “transplant”, “assisted migration”, “assisted colonization/colonisation”), and conservation or restoration contexts (“conservation”, “restoration”). Equivalent search strings were used in both databases, with syntax adapted to their specific requirements (i.e., TITLE-ABS-KEY in Scopus and Topic [TS] in Web of Science). Wildcards were applied where appropriate to capture term variations (e.g., translocat*, reintro*, transplant*), as follows:

- The search string used in Scopus was: (lichen OR “lichenized fungi”) AND (translocation OR relocation OR reintroduction OR transplant OR translocat* OR reintro* OR “assisted migration” OR “assisted colonization” OR “assisted colonisation”) AND (conservation OR restoration).
- In Web of Science, the following query was applied: TS=(lichen OR lichens OR “lichenized fungi”) AND TS = (translocat* OR reintro* OR transplant* OR relocation OR “assisted migration” OR “assisted colonization” OR “assisted colonisation”) AND TS = (conservation OR restoration).
- In Google Scholar, a more restrictive query was used due to database limitations: (“lichen translocation” OR “lichen transplantation” OR “lichen reintroduction”) AND (conservation OR restoration). The approach ensured that retrieved records were specifically focused on active lichen translocation practices, minimizing the inclusion of studies in which lichens were only marginally mentioned.

All records retrieved from the two databases were merged and duplicates were removed prior to screening. The selection process was carried out based on title and abstract screening, followed by full-text assessment of potentially relevant articles.

Searches were not restricted by substrate at the query stage; however, only studies dealing with epiphytic lichens were retained, while studies focusing exclusively on epilithic or epigeic lichens were excluded during the selection process.

The temporal scope of the review extended from 1970 to 2025. In addition to database searches, the reference lists of all selected papers were examined to identify further relevant studies on lichen translocation. For each selected study, information was extracted on publication year, country, bioregion, lichen taxa, growth forms, and conservation status, type and purpose of translocation, type of propagule, fixation technique, monitoring duration, and number of translocated samples, source population characteristics and site-selection criteria, indicators of translocation success, reported outcomes, and problems encountered.

Names and bio-ecological characteristics of the taxa followed ITALIC 8.0 [53]. Biome attribution was based on a global terrestrial ecoregion classification framework [58].

The results were synthesized through a descriptive quantitative mapping of the literature, combined with a qualitative critical analysis of methodological approaches and reported limitations. No meta-analysis was performed due to the high heterogeneity of the available data.

Generative artificial intelligence (GenAI) was used in this paper to assist in data collection (to identify further relevant studies dealing with lichen translocation in the selected papers) and for language editing. The authors take full responsibility for the content.

## 5. Conclusions

This scoping review provides a critical and up-to-date synthesis of conservation-oriented translocation practices applied to epiphytic lichens, highlighting their potential, limitations, and major knowledge gaps. The analysis of available case studies shows that translocation has been used predominantly in experimental contexts or for population reinforcement, with a strong methodological bias toward thallus fragments as propagules and with success most often assessed through short-term survival metrics.

Overall, the evidence indicates that translocation can support the initial persistence of epiphytic lichens when favorable environmental conditions are met and when fixation techniques ensure adequate stability of the translocated material. However, current data are insufficient to demonstrate its effectiveness as a long-term conservation strategy. High early survival may create an “illusion of success” if not supported by continued growth, reproduction, recruitment, and population expansion. Long-term monitoring, where available, reveals progressive attrition over time and provides little evidence of secondary colonization or demographic self-sustainability. Key indicators such as reproduction, recruitment, secondary dispersal, and population self-maintenance are rarely addressed, and monitoring schemes are frequently too short to capture meaningful demographic processes. In particular, sexual regeneration, recruitment from ascospores, and maintenance of genetic recombination and adaptive potential are almost never evaluated, despite their importance for long-term resilience.

An additional and critical limitation concerns the spatial scale at which epiphytic lichen translocation has been applied and evaluated to date. The vast majority of reviewed studies operate at local or site-specific scales, often restricted to single populations, forest patches, or narrowly defined microhabitats. While such interventions may be effective in mitigating local losses or reinforcing remnant populations, there is little evidence supporting their effectiveness at landscape or regional scales. In the absence of parallel conservation of environmental quality and habitat continuity over broader areas, translocation risks producing spatially confined and potentially transient effects, with limited relevance for the long-term conservation of epiphytic lichen diversity.

The main drivers of failure consistently emerge as technical constraints, particularly early sample loss and inadequate fixation, together with mismatches between donor and recipient microhabitats. Although biotic interactions and extreme climatic events appear to play a comparatively minor or poorly documented role in the available literature, their cumulative effects over longer timescales remain insufficiently explored. Taken together, these findings suggest that, under current knowledge, translocation should be regarded as a tool complementary to in situ conservation measures, such as habitat protection and restoration, reduction in environmental pressures, and maintenance of forest continuity, rather than as a stand-alone or substitute solution for epiphytic lichen conservation.

Future conservation efforts should therefore adopt more integrated approaches, including finer ecological characterization of target species, systematic comparison of different propagule types, explicit consideration of spatial scale, and, crucially, long-term monitoring frameworks designed to assess population self-sustainability. Greater conceptual clarity in defining “success” and explicit distinction between individual persistence and population-level viability will be essential to determine the real contribution of translocation to epiphytic lichen conservation under ongoing environmental change. Incorporating explicit vs conservation benefit analyses into translocation planning will be essential to avoid misinterpretation of short-term outcomes and to ensure that conservation efforts provide real and lasting ecological benefits.

## Figures and Tables

**Table 1:** Occurrence of lichen species across the analyzed papers (species listed in alphabetical order). References marked with “*” indicate studies in which the species is reported as nationally threatened.

Lichen Species	Articles
*Allographa sterlingiana* (E.A. Tripp and Lendemer) Lücking & Kalb	[37]
*Bryocaulon pseudosatoanum* (Asahina) Kärnefelt	[38] *
*Crocodia aurata* (Ach.) Link	[39]
*Dendriscosticta hookeri* (Trevis.) Moncada & Lücking	[40]
*Dolichousnea longissima* (Ach.) Articus	[40]
*Erioderma pedicellatum* (Hue) P.M.Jørg.	[41] *
*Evernia divaricata* (L.) Ach.	[42] *
*Hypogymnia flavida* McCune & Obermayer	[40]
*Hypotrachyna cirrhata* (Fr.) Divakar et al.	[40]
*Hypotrachyna virginica* (Hale) Hale	[37] *
*Lepraria finkii* (B. de Lesd.) R.C. Harris	[37]
*Lepraria lanata* Tønsberg	[37]
*Leptogium hibernicum* M.E. Mitch. ex P.M. Jørg.	[17] *
*Leptogium saturninum* (Dicks.) Nyl.	[17,43]
*Lobaria pindarensis* Räsänen	[40]
*Lobaria pulmonaria* (L.) Hoffm.	[14,38,44] * [35,36,45,46,47,48,49,50]
*Lobaria retigera* (Bory) Trevis.	[40]
*Menegazzia terebrata* (Hoffm.) A. Massal.	[43] *
*Parmotrema crinitum* (Ach.) M. Choisy	[44] *
*Parmotrema reticulatum* (Taylor) M. Choisy	[40]
*Platismatia norvegica* (Lynge) W.L. Culb. & C.F. Culb.	[42] *
*Ramalina conduplicans* Vain.	[40]
*Ramalina dilacerata* (Hoffm.) Hoffm.	[42] *
*Ricasolia amplissima* (Scop.) De Not.	[33,34]
*Sticta fuliginosa* (Hoffm.) Ach.	[43] *
*Sticta sylvatica* (Huds.) Ach.	[44] *
*Sulcaria isidiifera* Brodo	[51] *
*Sulcaria sulcata* (Lév.) Bystrek ex Brodo & D. Hawksw.	[40]
*Teloschistes flavicans* (Sw.) Norman	[52] *
*Usnea angulata* Ach.	[37]

**Table 2:** Quantitative assessment of lichen translocation success by material type, including only papers providing numerical estimates of survival, retention, establishment or growth in relation to the number of translocated samples and monitoring time since translocation.

Id	Translocated Material	Primary Success Criterion	Reported Success
	WHOLE THALLI		
	species (n)	n (%) of surviving thalli or cm—time ^1^	type
[33,34]	*Ricasolia amplissima* (14)	10 (71%)—1 yr, 10 (71%)—10 yrs; 6 (43%)—20 yrs	Long-term survival + growth
[35,36]	*Lobaria pulmonaria* (1120)	89%—20-25 mos; 23%—14 yrs	Thallus survival
[37]	*Usnea angulata* (16)	~1.7 cm linear increase—1 yr	Thallus growth
[38]	*L. pulmonaria* (26)	26 (100%)—1 yr	Thallus survival
[40]	9 spp. (540; 60/species)	(78%, aggregated)—3 yrs	Thallus survival + physiology
[41]	*Erioderma pedicellatum* (54)	54 (100%)—6 mos	Thallus growth + physiology
[49]	*L. pulmonaria* (30)	29 (97%)—4 yrs	Thallus survival
[49]	*L. pulmonaria* (30)	26 (87%)—4 yrs	Thallus survival
[51]	*Sulcaria isidiifera* (24)	22 (92%)—3.5 yrs	Thallus survival
	THALLUS FRAGMENTS		
	species (n)	n (%) of surviving thalli or cm—time ^1^	type
[17]	*Leptogium hibernicum* (125)	12%—4 yrs	Fragment retention
[17]	*Leptogium saturninum* (100)	19%—4 yrs	Fragment retention
[37]	*Allographa sterlingiana* (36), *Hypotrachyna virginica* (36), *Lepraria lanata* (9)	≈1% overall (aggregate report)—6-9 mos	Fragment retention
[37]	*Lepraria finkii* (5/6 per fixing methods, multiple test)	0–80% depending on method—6-9 mos	Fragment retention
[42]	*Evernia divaricata* (50 per fixing method, multiple test)	85–98% depending on method—1 yr	Fragment retention + growth
[42]	*Ramalina dilacerata* (50 per fixing method, multiple test)	85–98% depending on method—1 yr	Fragment retention + growth
[43]	*Menegazzia terebrata* (100)	40-45% retained—2 mos, <20% retained—16 mos	Fragment retention
[44]	*L. pulmonaria* (?)	≈0.1 cm/yr linear increase in ~50% retained—4 yrs	Adult lobes growth
[50]	*L. pulmonaria* (80)	10-31% area increase in 44% of fragments	Thallus growth
	VEGETATIVE PROPAGULES (ISIDIA/SOREDIA)		
	species—type ^1^ (n)	n (%) of retained/established propagules—time ^1^	type
[14]	*L. pulmonaria*—IsS (200)	~100 (50%)—2 mos, 58 (29%)—16 mos	Retention
[39]	*Crocodia aurata*—So packets (100)	13 (13%) developing lobes—2 yrs	Establishment
[42]	*Platismatia norvegica—* Is (240 across 48 microsites)	~10–15% of microsites with microthalli—1 year	Establishment (microsite-based)
[43]	*Sticta fuliginosa*—So (100)	52 (52%)—2 mos, 29 (29%)—16 mos	Retention
[43]	*L. saturninum*—Is (100)	46 (46%)—2 mos, 19 (19%)—16 mos	Retention
[44]	*L. pulmonaria*—IsS (?)	2/5 sites regeneration	Site-level regeneration
[44]	*Parmotrema crinitum*—Is (? per fixing methods, multiple test)	1.3–14% depending on method—2–6 mos	Retention

^1^ 1 yr = year; yrs = years; mo = month; mos = months; IsS = isidioid soredia; So = soredia; Is = isidia.

## Data Availability

No new data were created or analyzed in this study.
